# A Snapshot Survey of Uterotonic Administration Practice During Cesarean Section: Is There a Difference Between the Attitudes of Obstetricians and Anesthesiologists?

**DOI:** 10.3390/medicina61020253

**Published:** 2025-02-01

**Authors:** Nuray Camgoz Eryilmaz, Selin Erel, D. Berrin Gunaydin

**Affiliations:** Department of Anesthesiology and Reanimation, Gazi University School of Medicine, 06500 Ankara, Turkey; selinerel@yahoo.com (S.E.); gunaydin@gazi.edu.tr (D.B.G.)

**Keywords:** uterotonic, oxytocin, cesarean section, postpartum hemorrhage, uterus, survey

## Abstract

*Background and Objectives*: We aimed to evaluate the current uterotonic administration practices among anesthesiologists and obstetricians and gynecologists (OBGYNs) during cesarean section (CS), focusing on variations in approaches for low- and high-risk postpartum hemorrhage (PPH) cases. The objective was to identify key differences and provide evidence that could contribute to the development of standardized national protocols for uterotonic usage. *Materials and Methods*: A snapshot online survey was employed between October 2021 and January 2022 and distributed to anesthesiologists and OBGYNs from university-affiliated, government, and private hospitals across Turkey, consisting of 23 questions addressing demographic data, institutional PPH rates, first-line uterotonic choices, administration methods, and dose adjustments for low- and high-risk PPH cases. Specific questions also targeted uterotonic usage in the presence of comorbidities such as pre-eclampsia and cardiac disease. *Results*: There were 204 responses (54% anesthesiologists and 46% OBGYNs) out of 220, yielding a response rate of 92.7%. Oxytocin was the most common first-line uterotonic for CS with low-risk PPH (99.1% of the anesthesiologists and 96.8% of the OBGYNs). In total, 60% of the anesthesiologists favored an intravenous (IV) bolus followed by infusion, while 56.4% of the OBGYNs preferred IV infusion alone (*p* < 0.001). For CS with high-risk PPH, approximately half of the participants reported increases in oxytocin dose, while 26.4% of the anesthesiologists and 20.2% of the OBGYNs opted for combined oxytocin and carbetocin use. During intrapartum CS, 69.1% of anesthesiologists and 77.7% of OBGYNs reported no change in dose. However, 11.8% of the anesthesiologists indicated combining oxytocin and carbetocin (*p* < 0.05). In managing pre-eclampsia and cardiac disease, the anesthesiologists were likely to reduce uterotonic doses (15.5%) and avoid methylergonovine (35.5%) compared to the OBGYNs, who reduced doses less frequently (4.3%), but 79.8% of the OBGYNs avoided methylergonovine (*p* < 0.001). *Conclusions*: There was considerable variability in uterotonic administration practices between the anesthesiologists and OBGYNs.

## 1. Introduction

The debate on either standard or rational protocols for the administration of uterotonics in parturients having low or high risk for postpartum hemorrhage (PPH) during cesarean section (CS) is still continuing. Since there is a worldwide variability in oxytocin use during CS in clinical practice, the attitudes of obstetricians and/or anesthesiologists towards uterotonic practices have been evaluated via surveys in a number of countries, like Australia, New Zealand, Israel, Canada, and the UK [1,2,3,4]. Uterotonic usage during elective CS was first determined via the survey conducted in Australia and New Zealand in 2021, and there was a significant variation in the oxytocin use among members of the Obstetric Anesthesia Special Interest Group [1]. According to the Israeli survey, oxytocin practices significantly varied between obstetricians and anesthesiologists in primary CS, while there was no difference in repeat CS. Additionally, the aggressive approaches of administering an IV bolus of 5 or 10 international units (IUs) with or without methergine as a second-line uterotonic management were reported [2]. Meanwhile, the Canadian survey reported that the first-line agent for vaginal delivery, in majority, was oxytocin (94%), whereas carbetocin was preferred at a very low rate (6%). However, for women undergoing CS at low risk for PPH, the rates of oxytocin and carbetocin were reported to be 66% and 34%, respectively. Regarding the women scheduled for CS with high risk of PPH, physicians reported initial use of both oxytocin (60%) and carbetocin (40%) [3]. The UK survey revealed that the majority of the respondents used initial doses of 5 IU, while the rest (5.3%) administered doses of <5 IU for elective CS, with a broad range of administration methods [4]. Besides uterotonic drugs in the management of placenta accreta spectrum (PAS), which has a high risk of PPH, hemostatic procedures, including modified one-step conservative uterine surgery (MOSCUS), have been presented as a new successful approach versus cesarean hysterectomy [5].

All these major surveys concluded that there is a need for national protocols due to the wide variation and lack of strong evidence to guide this practice. Therefore, we aimed to investigate the current situation by collecting data via the present countrywide survey to present the uterotonic preferences of obstetricians and/or anesthesiologists during CS to prepare standard/rational protocols that would potentially contribute to the literature in this clinically important practice area.

## 2. Materials and Methods

This study was structured as a prospective observational investigation utilizing a targeted online survey approach. Institutional Ethics Committee approval was obtained from the Gazi University School of Medicine (Number: E-77082166-604.01.02-196986; Date: 21 October 2021). This study was registered at https://inclinicaltrials.com/cesarean-section-complications/NCT05089721/details/ (accessed on 1 October 2021 and verified on October 2021). The survey targeted anesthesiologists and obstetricians and gynecologists (OBGYNs). For data collection, 220 email addresses were sourced from the websites of university, government, private, and teaching hospitals. Afterward, e-mails with a questionnaire were sent to OBGYNs and anesthesiologists, and in cases of unavailable or inaccessible email addresses, these individuals were contacted via WhatsApp notifications to request the return of their completed questionnaires. Inclusion criteria were based on the voluntary participation of the clinicians in this survey, and individuals who chose not to participate were excluded. The inclusion criteria for this study were being a specialist anesthesiologist or OBGYN and participating voluntarily. The exclusion criterion was a questionnaire that was filled out incompletely.

An invitation letter detailed the study objectives, accompanied by a link to the questionnaire hosted on the “Survey Monkey” platform (Surveymonkey.com, Palo Alto, CA, USA). Consent to participate was inferred if the contacted person agreed to respond to the questionnaire, which consisted of five sections, including 23 questions prepared in Turkish, based on international/national surveys [1,2,3,4,6] (Table 1). Then, it was translated into English for the publication of this article (See Appendix A). The survey was conducted during a three-month snapshot window between 21 October 2021 and 21 January 2022. The first 3 out of 23 questions were related to demographic information, while questions 4–5 questioned the CS (where the final indication was made by OBGYNs) and PPH rates of the centers. Questions 6 to 13 inquired about the initial choice of uterotonic agent during CS, as well as preferences for uterotonic agents in case of either a low or high risk of PPH. These questions specifically addressed the usage of oxytocin and/or carbetocin in parturients having either low or high risk of PPH. Question 14 inquired about the uterotonic preferences and dose adjustments during CS where labor had commenced. Questions 15–17 addressed second-line uterotonic agent preferences. Questions 18–23 explored uterotonic preferences and dose regimens in pre-eclampsia (new onset preeclampsia BP > 140/90 mmHg) and cardiac disease (valve disease and/or congenital) with accompanying PPH.

### Statistical Analysis

A total of 220 participants who responded to the e-mail were included through a simple random sampling technique that targeted at least a 70% response rate based on a previous major survey [4]. With a population size of 220 participants, a 95% confidence interval, and a 5% marginal error, the sample size was calculated as 141 participants. The Statistical Package for the Social Sciences (SPSS) 22.0 (IBM SPSS, Inc., Chicago, IL, USA, 2020) statistical software was used for statistical analysis, and the results were presented. After descriptive statistics, categorical variables were expressed as n or percent where appropriate. A Pearson chi-square test was used to compare categorical variables. A *p* value less than 0.05 was considered as statistically significant.

## 3. Results

**Figure 1:**
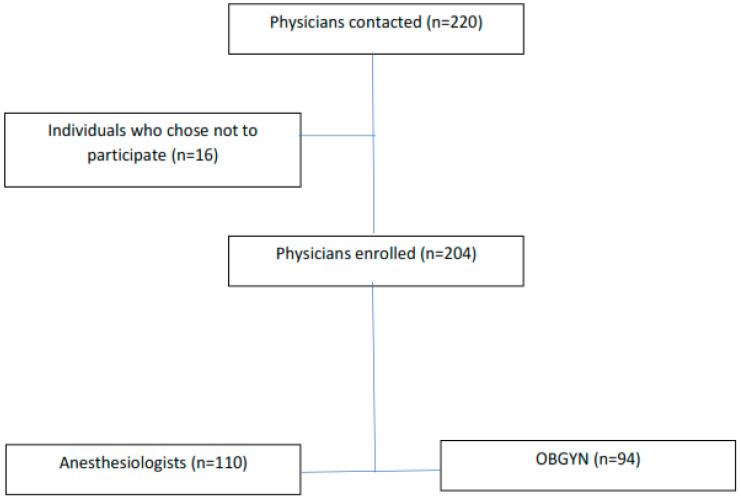
Flowchart.

A total of 204 out of 220 clinicians responded. The response rate was 92.7%. Since participation in the study was voluntary, 16 individuals who chose not to participate were excluded. Among 204 respondents, 110 (54%) were anesthesiologists, and 94 (46%) were OBGYNs as shown in the flowchart (Figure 1).

Most of the participants were from state-affiliated hospitals (*p* < 0.016) with 10–20 years of practice experience in their specialties (*p* < 0.001) (Table 2). The majority of the participants (47.3% of the anesthesiologists and 63% of the OBGYNs) reported that the annual CS rate was greater than 30%. The PPH rate exceeding 3% was similar between the anesthesiologists and OBGYNs (18.2% and 17%). However, there was a considerable disparity in the reported rate of PPH less than 3% (76.6% of OBGYNs vs. 47.3% of anesthesiologists). Furthermore, a considerable percentage of anesthesiologists noted that insufficient information was provided about PPH rates, highlighting an important observation within the data (Table 2).

In CS with a low risk of PPH, oxytocin was the most common first-line uterotonic both by the anesthesiologists (99.1%) and OBGYNs (96.8%). Carbetocin preference was significantly lower compared to oxytocin (0.9% for anesthesiologists and 3.2% for OBGYNs).

Notably, 60% of the anesthesiologists preferred IV bolus followed by the infusion method, whereas 56.4% of the OBGYNs favored IV infusion, which was statistically significant (*p* < 0.001) (Table 3). The preferred method of administering carbetocin as the primary uterotonic during CS with a low risk of PPH via IV bolus using a slow bolus technique (slower than one minute) did not differ between the two specialties (Table 3). Regarding the preference for administering oxytocin and carbetocin concurrently via the IV bolus alongside oxytocin via IV infusion, there was no significant difference between the two specialties (Table 3).

In cases of CS with a high risk of PPH, almost 50% of anesthesiologists and 56.4% of the OBGYNs stated that they increased the dose of oxytocin (*p* > 0.05). Both anesthesiologists (26.4%) and OBGYNs (20.2%) reported using oxytocin and carbetocin together without increasing the dose (*p* > 0.05) (Table 4). When oxytocin was the first-choice uterotonic, 63.6% of anesthesiologists and 40.4% of OBGYNs preferred the IV bolus + infusion method, while 29.8% of OBGYNs and 10% of anesthesiologists preferred the IV infusion method (*p* < 0.002) (Table 4). However, when carbetocin alone and oxytocin + carbetocin were the first-choice uterotonics, there was no significant difference between the two specialties (*p* > 0.05) (Table 4).

For the question, “Does your uterotonic dose change in patients in labor during CS?” (intrapartum CS), 69.1% of anesthesiologists and 77.7% of OBGYNs answered that “it does not change”. Unlike OBGYNs, 11.8% of anesthesiologists reported using oxytocin and carbetocin together (*p* < 0.05) (Table 5).

In high-risk patients, such as those with preeclampsia and pregnant women with cardiac disease, there was a protocol change for using uterotonic agents according to the replies of the OBGYNs (84%) and anesthesiologists (70.9%) (*p* = 0.026). Regarding these attitudes, 15.5% of anesthesiologists reduced the doses of oxytocin and carbetocin, 35.5% of them did not use methylergonovine, and 21.8% of them reduced all uterotonic doses (*p* < 0.001). In contrast, the 79.8% of OBGYNs stated that they did not use methylergonovine, while 4.3% reduced uterotonic doses (*p* < 0.001) (Table 5).

In the treatment of PPH, 51.8% of anesthesiologists preferred the IV bolus oxytocin dose of less than 5 IU, while 36.2% of OBGYNs preferred 10 IU, showing a significant difference between clinicians (*p* < 0.05). As for the oxytocin infusion dose, 41.8% of anesthesiologists and 19.1% of OBGYNs preferred less than 20 IU, and 1.8% of anesthesiologists and 17% of OBGYNs preferred ≥40 IU, which was significantly different (*p* < 0.001). But there was no significant difference in those who preferred 20–30 IU versus 30–40 IU (Table 6). Both anesthesiologists and OBGYNs preferred to treat PPH with 50 μg of IV bolus carbetocin (*p* > 0.05) (Table 6). Notably, 97.9% of OBGYNs reported using misoprostol, while no anesthesiologists did (*p* > 0.001) (Table 6).

## 4. Discussion

In the present snapshot survey, the first-line uterotonic choice during both elective and emergency CS was found to be oxytocin with an overall rate of 98% by both OBGYNs and anesthesiologists. The oxytocin administration via IV bolus + infusion and infusion alone were preferred by anesthesiologists and OBGYNs, respectively. In cases of CS with a low risk of PPH, the administration methods for either carbetocin alone (IV slow bolus) or oxytocin plus carbetocin did not differ between physicians. However, in the treatment of PPH, the use of less than 5 IU of IV bolus oxytocin was significantly greater among the anesthesiologists compared to OBGYNs. There was a significant difference in the attitudes between anesthesiologists and OBGYNs, particularly in terms of oxytocin IV bolus and infusion regimens.

The beliefs and practice for uterotonic use during elective CS was surveyed among members of the Obstetric Anesthesia Special Interest Group in Australia and New Zealand; the response rate was 33% from an analysis of 279 completed reports. It was reported that oxytocin was routinely administered via the IV bolus as a first-line uterotonic as 5 IU (38%), as <5 IU (38%), 10 IU (10%), and carbetocin of 100 μg (13%) [1]. In the current survey, our response rate was 92.7%, which is quite high. Among the 204 respondents, 110 (54%) of them were anesthesiologists, and 94 (46%) of them were OBGYNs. In our initial survey, which was conducted between the years 2016–2018 by the obstetric anesthesia subcommittee of our national society, an oxytocin IV bolus of 5 IU followed by infusion of 20 IU/L was used as the routine protocol for the management of elective CS [6]. Hereby, we encountered that the protocol included oxytocin via IV infusion by OBGYNs, IV infusion + bolus (the effective dose 90% (ED90) or less than 5 IU) by the anesthesiologists, and carbetocin via IV slow bolus by anesthesiologists + OBGYNs. In case of using two uterotonics together, carbetocin (in bolus) + oxytocin (via infusion) was preferred similarly by the clinicians.

According to the Israeli survey, 391 out of 429 physicians responded; oxytocin practices significantly varied between obstetricians and anesthesiologists in primary CS, while no significant difference was observed in repeat CS. In addition to the reported aggressive approach of administering the IV bolus of 5 or 10 IU, increased treatment with methergine as a second-line uterotonic management was found [2]. Instead of categorizing CS as primary or repeat, we structured our survey similarly to the Canadian survey [3] by questioning whether CS has either a low or high risk of PPH, since PPH has been a major source of high maternal mortality rate (MMR). The MMR resulting from PPH in Turkey was 13.1 in 100,000 live births in 2019 [7].

In the Canadian survey, 33 out of 92 clinicians (obstetricians and anesthesiologists) where 24 out of 46 were from university affiliated hospitals. The response rate was 35%. The first-line agents for vaginal delivery were oxytocin (94%) and carbetocin (6%). As for women at low risk of PPH undergoing CS, 66% reported that oxytocin was a first-line uterotonic, while 34% reported carbetocin. Regarding CS at high risk of PPH, 60% of physicians reported oxytocin and 40% reported carbetocin. The second-line uterotonic use varied mainly based on perceived efficacy by the Society of Obstetricians and Gynecologists of Canada (SOGC) guidelines [3].

According to UK survey, which has a 72.9% response rate, the majority of the respondents were using an initial 5 IU dose, while the rest (5.3%) administered a dose <5 IU for elective CS. The administration of a 10 IU dose has ceased altogether. There has been a broad range of administration methods, particularly with infusions. Forty (26.8%) respondents stated a different oxytocin regimen following CS in severe preeclampsia, 72 (48.3%) in those with cardiac disease of New York Heart Association class 1–2, and 100 (66.7%) with class 3–4. [4]. The findings in these surveys highlighted differences in clinical approaches between OBGYNs and anesthesiologists while also underscoring the need to establish standardized guidelines supported by the recent literature to ensure consistent management practices.

Our results are consistent with previous surveys conducted in other countries [1,2,3,4].

In our national anesthesiology society’s survey including only 191 anesthesiologists, 69% of them stated that they preferred IV bolus + infusion, while 30% of them preferred oxytocin of 20 IU/1000 mL saline or Ringer’s lactate solution [6]. The current survey revealed that after delivery, the majority of OBGYNs (86.2%) preferred to use methylergonovine when compared to anesthesiologists (65.5%), while 25.5% of OBGYNs routinely used methylergonovine in addition to oxytocin and/or carbetocin. However, anesthesiologists (64.5%) preferred methylergonovine when compared to OBGYNs (43.6%) in case of uterine atony. Notably, almost all the specialists preferred the intramuscular administration route for methylergonovine as prescribed.

Bolus studies in determining the effectiveness of different doses of uterotonics demonstrated that 0.35 IU of oxytocin effectively achieved an adequate uterine tone in 90% of women 3 min after administration [8]. This finding provides a specific dosage that can reliably prevent uterine atony. Additionally, there was a ceiling effect for oxytocin dose >0.5 IU [9].

The reported benefits of oxytocin infusion after bolus administration compared to bolus-only regimens include reduced estimated blood loss, less need for blood transfusion, and less requirement for additional uterotonics. These findings highlight the potential advantages of incorporating infusion into uterotonic protocols during CS [10,11]. Oxytocin has been commonly administered via rapid, rather than slow, infusion to initiate and further maintain uterine tone. Because of the need for optimizing the uterotonic dose, which is essential to minimize adverse effects, a “rule of threes” algorithm, rather than continuous infusion of oxytocin, during elective CS has become popular [12].

The ED90 dose of oxytocin infusion to obtain the satisfactory uterine tone at an initial assessment 4 min after delivery was found to be 0.29 IU min^−1^ or 17.4 IU h^−1^. Later, it was reported that ED90 dose for oxytocin was 0.27 IU min^−1^ or 16.2 IU h^−1^ to initiate and maintain an adequate uterine tone. These findings suggested that a standardized approach to oxytocin infusion rates can ensure consistent outcomes in terms of uterine tone and minimize the risk of PPH [13,14]. It has been reported that a comparison of high-rate infusion of oxytocin (15 IU h^−1^) with low-rate infusion (2.5 IU h^−1^) during elective CS neither enhanced the uterine tone nor decreased the rate of PPH. According to the consensus guideline on the use of uterotonic agents during CS, the administration 1 IU of oxytocin in the IV bolus followed by infusion at 2.5–7.5 IU h^−1^ was recommended rather than that of outdated traditional 5 or 10 IU IV bolus doses [11,15,16,17,18,19,20,21].

Anesthesiologists in the UK used to administer 5 IU IV bolus as a routine practice, according to the Royal College of Obstetricians and Gynecologists (RCOG)’s guideline in 2016 [15]. Although RCOG has not recently launched an updated guideline, later, in 2021, the favored oxytocin dose was <5 IU, and the carbetocin dose was 100 μg in the Australian and New Zealand guidelines [1]. In contrast to the Canadian survey’s findings, we documented the first-choice uterotonic for oxytocin (98%) and for carbetocin (2%) at a low risk of PPH when undergoing CS. The higher rate of carbetocin administration in Canada (66% for oxytocin and 34% for carbetocin) compared to our survey may be attributed to the participants referencing the SOGC guidelines. While the SOGC guidelines recommended carbetocin 100 μg as the first-line medication [22], based on the ED90 dose of carbetocin, which was 14.8 µg, carbetocin doses as low as 20 μg were recommended [22,23]. In our current survey, respondents administered carbetocin via the slow IV bolus route together with oxytocin infusion.

The Australian survey revealed that 58% preferred an oxytocin bolus plus infusion at high doses, such as 40 IU. In the Canadian survey, oxytocin infusion either alone or followed by a bolus was used. In Turkey, we observed that while OBGYNs preferred the infusion-only method, anesthesiologists preferred the bolus + infusion method.

Several studies have indicated that lower doses of oxytocin may be sufficient to establish an adequate uterine tone during elective cesarean delivery compared to traditional recommendations [8,9]. However, the optimal oxytocin dosage can vary depending on the clinical scenario. For instance, pregnant individuals undergoing CS due to labor arrest following oxytocin augmentation may require nearly nine times the standard dose of oxytocin to achieve the adequate uterine tone [24]. Similarly, pregnant individuals with a body mass index (BMI) ≥40 kg/m^2^ may require approximately twice the amount of oxytocin compared to those with a BMI <40 kg m^2^. The estimated ED90 doses for twin pregnancies to achieve the adequate uterine tone for oxytocin were found to be 4.38 IU (95% CI, 3.68–4.86 IU) and 3.41 IU (95% CI, 2.83–3.98 IU), respectively. They recommended an initial bolus dose of 5 IU for elective CS under neuraxial anesthesia in these particular patients [25].

During intrapartum CS, multiple pregnancies, previous PPH history, polyhydramnios, which carry a high risk of PPH, and an oxytocin bolus dose of 5 IU were a routine UK practice, whereas the preferred infusion regimen was 7.5–10 IU h^−1^. In our survey, almost half of the respondents from both specialists chose to increase the dose of oxytocin. Approximately 20–25% of respondents used oxytocin and carbetocin together without an increase in dose. The Canadian guideline recommends 100 μg IM of carbetocin if a vaginal delivery has a risk factor for PPH, but, interestingly carbetocin recommendation for CS having high risk for PPH lacks in the guideline. However, the Canadian survey reported carbetocin use at a rate of 40% for CS with high-risk PPH [3]. In our institution, we have been administering IV slow bolus of carbetocin 50 µg and 100 µg for elective CS having low and high risk PPH, respectively. An emerging trend to use carbetocin (100 µg over 1 min) rather than oxytocin as the first-line uterotonic in some units was supported by the recommendation of SOCG for using carbetocin for elective CS [22].

Intrapartum CS represents an increased risk associated with prolonged labor, oxytocin receptor desensitization, and uterine overdistension. However, none of the major surveys, except the consensus statement, investigated this issue [1,2,3,4,11]. Therefore, we decided to make it clear in our questionnaire. Our results showed that 69.1% of anesthesiologists and 77.7% of OBGYNs reported no change in uterotonic dosing during intrapartum CS. This finding might be contradictory to the international consensus statement [11], which favors increasing oxytocin doses during intrapartum CS. In our survey, anesthesiologists (11.8%) reported using oxytocin plus carbetocin, while only 1.1% of OBGYNs’ choice was a combination of oxytocin and carbetocin. This suggests a more proactive approach by anesthesiologists to mitigate uterine atony risks through combination therapy.

The combined use of oxytocin and carbetocin was a notable preference among anesthesiologists, particularly at reduced doses for high-risk patients, such as those with preeclampsia or cardiac disease. In our study, 15.5% of anesthesiologists reported dose reductions for both agents in high-risk scenarios. Carbetocin’s prolonged action and reduced need for additional infusions make it a valuable option during intrapartum CS. Obstetricians overwhelmingly avoided the use of methylergonovine (79.8%). Since methylergonovine’s potent vasoconstrictive properties can exacerbate hypertension or cardiac complications, avoidance of methylergonovine in such patients aligns with best practices [11].

The survey also highlighted differences in uterotonic preferences in the treatment of PPH. While 51.8% of anesthesiologists favored IV bolus doses of oxytocin below 5 IU for PPH treatment, 36.2% of obstetricians preferred 10 IU. Anesthesiologists commonly utilized infusion doses below 20 IU (41.8%), whereas obstetricians more frequently administered doses exceeding 40 IU (17%). Misoprostol was widely used by obstetricians (97.9%) but not by anesthesiologists.

We believe that the different approaches of OBGYNs and anesthesiologists during CS are due to their different priorities: obstetricians often target a strong uterine tone, using higher uterotonic doses to prevent bleeding, while anesthesiologists focus on maternal hemodynamic stability, preferring optimal doses or alternative methods to avoid side effects such as hypertension.

In our survey, we observed the IV bolus + infusion method by both physician groups. Despite it seeming reasonable to give a higher dose for parturients with a high risk of PPH, high rates of hypotension, tachycardia, and dysrhythmia can occur. Our survey’s results were similar and mostly consistent with the literature, except a lower rate of carbetocin preference than that of the Canadian survey.

One of the limitations of this study could be the population of the respondents chosen. Since obstetric anesthesia has not yet become a subspecialty in the country, anesthesiologists who responded to the survey were not practicing only obstetric anesthesia. Another limitation of this survey could be not questioning the role of tranexamic acid use along with uterotonics. Recently, the role of tranexamic acid in reducing calculated blood loss during cesarean delivery was investigated, but it did not show a significantly lower risk of a composite outcome of maternal death or blood transfusion compared to a placebo [26]. Further survey studies can be planned to investigate the hemostatic agents used in the management of PPH together with uterotonics.

## 5. Conclusions

Basically, oxytocin remained the predominant first-line uterotonic, with variations in dose, administration methods, and management strategies. Since most of the surveys, including ours, until now have questioned carbetocin use limitedly, upcoming protocols might favor effective insights for better maternal outcomes.

## Figures and Tables

**Table 1:** Survey description.

Questions 1–3	Demographics
Questions 4–5	Rate of CS and PPH
Questions 6–13	Initial choice uterotonic for CS (low or high PPH)
Questions 14	Uterotonic for intrapartum CS
Questions 15–17	Second line uterotonics
Questions 18–23	Uterotonic preference for comorbidities and PPH

**Table 2:** Institutional and professional data of survey respondents and rates of CS and PPH reported by the clinicians [n (%)].

	**Total** **n = 204**	**Anesthesiologists** **n = 110**	**OBGYN** **n = 94**	***p***
Workplace				
State hospital	88 (43.1)	55 (50) *	33 (35.1)	0.016
University hospital	83 (40.7)	44 (40)	39 (45.1)	
Private hospital	33 (16.2)	11(10)	22 (23.4)	
Duration of clinical practice (years)				
<5	49 (24.0)	16 (14.5) *	33 (35.1)	<0.001
5–10	51 (25.0)	30 (27.3)	21 (22.3)	
10–20	67(32.8)	49 (44.5) *	18 (19.1)	
>20	37 (18.1)	15 (13.6)	22 (23.4)	
CS Rate				
<10%	11 (5.4)	8 (7.3)	3 (3.2)	0.03
10–20%	35 (17.2)	23 (20.9)	12 (12.8)	
21–30%	43 (21.1)	27 (24.5)	16 (17.0)	
>30%	115 (56.4)	52 (47.3) *	63 (67.0)	
PPH Rate				
<3%	124 (60.8)	52 (47.3) *	72 (76.6)	<0.001
>3%	36 (17.6)	20 (18.2)	16 (17.0)	
Unknown	44 (21.6)	38 (34.5) *	6 (6.4)	

CS: Cesarean section; PPH: postpartum hemorrhage; OBGYN: obstetrician and gynecologist; * *p* < 0.05 between anesthesiologists and OBGYNs; Pearson chi-square test.

**Table 3:** Preferences for uterotonics for CS at low risk of PPH [n (%)].

		Anesthesiologist n = 110	OBGYN n = 94	*p*
First-choice uterotonic	Oxytocin	109 (99.1%)	91 (96.8%)	0.241
	Carbetocin	1 (0.9%)	3 (3.2%)	
Route for oxytocin	IM oxytocin	5 (4.5%)	2 (2.1%)	<0.001
	IV infusion	26 (23.6%) *	53 (56.4%)	
	IV bolus + infusion	66 (60%) *	20 (21.3%)	
	IV bolus, (>1 min)	5 (4.5%)	10 (10.6%)	
	IV bolus, (<1 min)	8 (7.3%)	9 (9.6%)	
Route for carbetocin	IM carbetocin	10 (9.1%)	11 (11.7%)	>0.05
	IV rapid bolus (<1 min)	6 (5.5%)	7 (7.4%)	
	IV slow bolus (>1 min)	33 (30%)	29 (30.9%)	
Route for oxytocin + carbetocin	Oxytocin + carbetocin bolus	5 (4.5%)	6 (6.4%)	>0.05
	IV carbetocin bolus + IV oxytocin infusion	24 (21.8%)	27 (28.7%)	
	Oxytocin bolus + carbetocin bolus + oxytocin infusion	23 (20.9%)	14 (14.9%)	

CS: Cesarean section; PPH: postpartum hemorrhage; OBGYN: obstetrician and gynecologist; IV: intravenous; IM: intramuscular; * *p* < 0.05 between anesthesiologists and OBGYNs; Pearson chi-square test.

**Table 4:** Preference for uterotonics in CS at high risk of PPH [n (%)].

		Anesthesiologist n = 110	OBGYN n = 94	*p*
Increase dose	Only oxytocin	55 (50%)	53 (56.4%)	>0.05
	Only carbetocin	2 (1.8%)	1 (1.1%)	>0.05
	Choose Oxytocin + carbetocin	29 (26.4%)	19 (20.2%)	>0.05
	Both oxytocin and carbetocin	7 (6.6%)	8 (8.5%)	>0.05
Route of oxytocin	IM oxytocin	3 (2.7%)	1 (1.1%)	>0.05
	IV rapid bolus (<1 min)	13 (11.8%)	14 (14.9%)	>0.05
	IV slow bolus (>1 min)	13 (11.8%)	13 (13.8%)	>0.05
	IV infusion	11 (10%) *	28 (29.8%)	0.002
	IV bolus + infusion	70 (63.6%) *	38 (40.4%)	0.002
Route for carbetocin	IM carbetocin	8 (7.3%)	8 (8.5%)	>0.05
	IV bolus, rapid bolus (faster than 1 min)	10 (9.1%)	16 (17%)	>0.05
	IV bolus, slow bolus (slower than 1 min)	33 (30%)	28 (29.8%)	>0.05
If the first choice is oxytocin + carbetocin at high-risk CS for PPH, what is the preferred route?	Oxytocin + carbetocin Bolus	3 (2.7%)	6 (6.4%)	>0.05
	IV carbetocin bolus + IV oxytocin infusion	17 (15.5%)	26 (27.7%)	>0.05
	Oxytocin bolus + carbetocin bolus+ oxytocin infusion	38 (34.5%)	25 (26.6%)	>0.05

CS: Cesarean section; PPH: postpartum hemorrhage; OBGYN: obstetrician and gynecologists; IV: intravenous; IM: intramuscular; * *p* < 0.05 between anesthesiologists and OBGYNs; Pearson chi-square test.

**Table 5:** Preference for uterotonic in intrapartum and high-risk CS [n (%)].

Questions	Response Options	Anesthesiologist n = 110	OBGYN n = 94	*p*
Uterotonic dose change during intrapartum CS	Increase oxytocin	19 (17.3%)	20 (21.3%)	>0.05
	Oxytocin + carbetocin	13 (11.8%) *	1 (1.1%)	0.01
	Increase oxytocin and carbetocin doses	2 (1.8%)	0 (0%)	>0.05
	No change	76 (69.1%)	73 (77.7%)	>0.05
Route for methylergonovine	IV	9 (8.2%)	6 (6.4%)	>0.05
	IM	89 (80.9%)	75 (79.8%)	>0.05
	Both of them	12 (10.9%)	13 (13.8%)	>0.05
Protocol change for uterotonics choice in high-risk patients (preeclampsia, pregnant women with cardiac disease)	Yes/No	78 (70.9%)/32 (29.1%) *	79 (84%)/15 (16%)	0.026
If answer is yes	I use oxytocin and carbetocin by reducing their doses	17 (15.5%) *	0 (0%)	<0.001
	I do not use methylergonovine	39 (35.5%)	75 (79.8%)	<0.001
	I reduce the doses of all uterotonic agents	24 (21.8%)	4 (4.3%)	<0.001

CS: Cesarean section; PPH: postpartum hemorrhage; OBGYN: obstetrician and gynecologists; IV: intravenous; IM: intramuscular; * *p* < 0.05 between anesthesiologists and OBGYNs; Pearson chi-square test.

**Table 6:** Uterotonic preference in the treatment of PPH [n (%)].

		Anesthesiologist n = 110	OBGYN n = 94	*p*
Oxytocin bolus dose	<5 IU	57 (51.8%) *	19 (20.2%)	<0.001
	5 IU	28 (25.5%)	23 (24.5%)	>0.05
	5–10 IU	0 (0%)	1 (1.1%)	>0.05
	10 IU	18 (16.4%) *	34 (36.2%)	<0.001
	no bolus	7 (6.4%) *	17 (18.1%)	<0.001
Oxytocin infusion dose	<20 IU	46 (41.8%) *	18 (19.1%)	<0.001
	20–30 IU	45 (40.9%)	41 (43.6%)	>0.05
	30–40 IU	16 (14.5%)	18 (19.1%)	>0.05
	>40 IU	2 (1.8%) *	16 (17%)	<0.001
	I do not use	1 (0.9%)	1 (1.1%)	>0.05
Carbetocin IV bolus	<50 µg	12 (10.9%)	6 (6.4%)	>0.05
	50 µg	27 (24.5%)	24 (25.5%)	>0.05
	100 µg	20 (18.2%)	23 (24.5%)	>0.05
	> 100 µg	3 (2.7%)	1 (1.1%)	>0.05
	I do not use	48 (43.6%)	40 (42.6%)	>0.05
Misoprostol	Yes/No	37 (33.6%)/73 (66.4%) *	92 (97.9%)/2 (2.1%)	<0.001

OBGYN: obstetrician and gynecologists; PPH: postpartum hemorrhage; µg: microgram; IU: international unit; * *p* < 0.05 between anesthesiologists and OBGYNs; Pearson chi-square test.

## Data Availability

All the data generated and analyzed in this study are included in this published article.

## Abbreviations

The following abbreviations are used in this manuscript:

CS	Cesarean Section
OBGYN	Obstetrician and Gynecologists
PPH	Postpartum Hemorrhage
IU	International Unit
MMR	Maternal Mortality Rate
SOGC	Society of Obstetricians and Gynecologists
RCOG	Royal College of Obstetricians and Gynecologists
ED90	The effective dose 90%

## Appendix A

**UTEROTONIC PRACTICE OF OBGYNS’ AND ANESTHESIOLOGISTS’ QUESTIONNAIRE** 

1. **Which type of hospital do you work?** 

- Private
- State
- University

2. **What is your area of expertise?** 

- Anesthesiologist
- Obstetrician-Gynecologist

3. **How long is your professional experience?** 

- <5 years
- 5–10 years
- 10–20 years
- >20 years

4. **What is the annual cesarean section (CS) rate among all deliveries in your hospital?** 

- <10%
- 10–20%
- 21–30%
- >30%

5. **What is your annual estimated postpartum hemorrhage (PPH) rate in your hospital?** 

- <3%
- >3%
- I don’t know

6. **What is your first choice of uterotonic agent for CS?** 

- Oxytocin
- Carbetocin
- Oxytocin+carbetocin

7. **If oxytocin is your first choice for CS with low risk PPH, which route do you prefer to use?** 

- IM oxytocin
- IV bolus, rapid push (faster than 1 min)
- IV bolus, slow push (slower than 1 min)
- IV infusion
- IV bolus + infusion

8. **If carbetocin is your first choice for CS with low risk for PPH, which route do you prefer to use?** 

- IM carbetocin
- IV bolus, rapid push (faster than 1 min)
- IV bolus, slow push (slower than 1 min)

9. **If oxytocin + carbetocin is your first choice for CS with low risk for PPH, which route do you prefer to use?** 

- Oxytocin+carbetocin bolus
- IV carbetocin bolus + IV oxytocin infusion
- Oxytocin bolus + carbetocin bolus + oxytocin infusion

10. **Do you change your uterotonic and/or dose in CS with high risk PPH?** 

- Yes, I change and I increase the oxytocin dose
- Yes, I change and I increase the carbetocin dose
- Yes, I change and I use oxytocin and carbetocin together
- Yes, I change and I increase the oxytocin and carbetocin doses together
- No, I do not change any of them

11. **If your first choice is oxytocin in CS with high risk for PPH, which route do you prefer to use? ** 

- IM oxytocin
- IV bolus, rapid push (faster than 1 min)
- IV bolus, slow push (slower than 1 min)
- IV infusion
- IV bolus + infusion

12. **If your first choice is carbetocin in CS with high risk PPH, which route do you prefer to use?** 

- IM carbetocin
- IV bolus, rapid push (faster than 1 min)
- IV bolus, slow push (slower than 1 min)

13. **If your first choice is oxytocin + carbetocin in CS with high risk PPH, which route do you prefer to use?** 

- Oxytocin + carbetocin bolus
- IV carbetocin bolus + IV oxytocin infusion
- Oxytocin bolus + carbetocin bolus + oxytocin infusion

14. **Does your uterotonic dose change for parturients in labor during a CS (intrapartum cesarean section)?** 

- Yes, it changes, I increase the oxytocin dose
- Yes, it changes, I increase the carbetocin dose
- Yes, it changes, I use oxytocin and carbetocin together
- It varies and I increase the dose of oxytocin and carbetocin together
- No, it does not change

15. **Do you prefer to use methylergonovine after cesarean delivery?** 

- Yes
- No

16. **If your answer is yes, when/how do you prefer to use it?** 

- Routinely in addition to oxytocin and/or carbetocin
- In cases of bleeding
- In cases of uterine atony
- Other (please explain)

17. **What is your routine route of methylergonovine administration?** 

- Intravenous
- Intramuscular
- Both

18. **Does your protocol for using uterotonic agents change in high-risk patients (preeclampsia, and cardiac disease, etc.)?** 

- Yes
- No

1G. **If your answer is yes;** 

- I use oxytocin and carbetocin by reducing their doses
- I do not use methylergonovine
- I reduce the dose of all uterotonic agents
- I do not change

20. **What is your bolus oxytocin dose for PPH treatment?** 

- Under 5 international units
- 5 international units
- 5–10 international units
- 10 international units
- I do not use bolus

21. **What is your oxytocin infusion dose for PPH treatment?** 

- Under 20 international units
- 20–30 international units
- 30-40 international units
- 40 international units and above
- I do not use infusion

22. **What is your bolus carbetocin dose for PPH treatment?** 

- Under 50 micrograms
- 50 micrograms
- 100 micrograms
- Over 100 micrograms
- I do not use carbetocin bolus

23. **Do you use misoprostol for PPH treatment?** 

- Yes
- No
